# Cost‐Effectiveness of Computer‐Assisted Cytology in a Primary hrHPV‐Based Cervical Cancer Screening Programme

**DOI:** 10.1002/cam4.70299

**Published:** 2024-10-14

**Authors:** Ellen M. G. Olthof, S. Kaljouw, Folkert J. van Kemenade, Anne M. Uyterlinde, Inge M. C. M. de Kok

**Affiliations:** ^1^ Department of Public Health Erasmus MC University Medical Center Rotterdam Rotterdam The Netherlands; ^2^ Department of Pathology Erasmus MC University Medical Center Rotterdam Rotterdam The Netherlands; ^3^ Department of Pathology Amsterdam University Medical Center Amsterdam The Netherlands

**Keywords:** cervical cancer, cervical cancer screening, computer‐assisted cytology, computer‐assisted screening, hrHPV screening

## Abstract

**Background:**

Computer‐assisted screening (CAS) shows equal performance compared to manual screening, although results are heterogeneous. Furthermore, using CAS may save costs through a potentially increased screening productivity of technicians, therefore also offering a solution for temporary and structural capacity shortage. We evaluated the circumstances under which CAS will be cost‐effective compared to manual cytology triage in a primary HPV‐based cervical screening programme.

**Methods:**

Microsimulation model MISCAN‐Cervix was used to evaluate 198 different CAS scenarios with varying probabilities to detect cervical intraepithelial neoplasia grade 1 (CIN1) and CIN3 and cost reductions per test, compared to manual cytology triage. Cost‐effectiveness was evaluated by costs per (quality‐adjusted) life year ((QA)LY) gained.

**Results:**

CAS will be cost‐effective in all scenarios, except for the following combinations: (1) no cost reduction and an increased probability of detecting CIN1, (2) a cost reduction of €2 per test and an increased probability of detecting CIN1 from 4% onwards or (3) a cost reduction of €4 per test and an increased probability of detecting CIN1 from 6% onwards, compared to manual cytology triage. All CAS scenarios with any reduction in the probability of detecting CIN1 (i.e., increased CIN2+ specificity), or a reduction in costs from €6 per test onwards suggested a more cost‐effective strategy compared to manual cytology triage.

**Conclusion:**

As we based our analysis on a realistic range in costs and test performance, the implementation of CAS is likely to be cost‐effective. Our results can be used as a guideline to advise when to choose CAS instead of manual cytology triage.

## Abstract 2

Computer‐assisted screening (CAS) is an evident substitute for manual cytology screening as a triage test in a primary HPV‐based setting. This is the first study investigating under which circumstances CAS is cost‐effective in a primary HPV‐based screening programme. We showed that in case of increased CIN2+ specificity and cost reduction from €6 per test, CAS was more cost‐effective than manual cytology triage. So, implementing CAS is likely to be cost‐effective in primary HPV‐based screening, which can guide decision‐making.

**Abbreviations**
CAS: computer‐assisted screening
CC: clinician collected
CIN: cervical intraepithelial lesion
GP: general practitioner
hrHPV: high‐risk human papillomavirus

## 1 Background

The Netherlands has a primary high‐risk human papillomavirus (hrHPV) based cervical cancer screening programme with cytology triage for hrHPV‐positive women [1]. Currently, cytology is mostly manually performed (since July 2023 approximately 10%–20% is performed with computer‐assisted screening, CAS). Smears are examined by a lab technician (mainly prescreening and proposing a Bethesda classification) and jointly signed out with a pathologist. Extensive research has previously been conducted to evaluate the benefits of CAS compared to manual screening [2]. CAS systems use algorithms to select areas of individual cells and groups of cells in so‐called fields of view (FOV) of a thin‐layer specimen, for a cytotechnologist to review and determine the likelihood of abnormal cells on the slide [3]. In case of detection of abnormal cells, the slide will be referred to manual screening. If the 22 fields (according to the standard protocol) are deemed to be normal, the technician can limit the examination to those 22 selected fields and thus increase productivity by saving screen time in the rest of the slide. With large volumes, using CAS instead of manual screening can save time, personnel and costs.

Currently, two algorithm‐based CAS systems are approved by the Food and Drug Administration in the USA: ThinPrep Imaging System (Hologic Corporation, Marlborough, MA, USA) and FocalPoint‐Guided Screening Imaging System (BD Diagnostics). In addition, deep learning‐based systems (Genius by Hologic and the Datexim) have been recently introduced. Recently, the Dutch Health Council published advise concerning CAS for cervical cancer, recommending CAS if similar outcomes regarding test performance are observed in a Dutch pilot study, compared to manual screening [4]. Previous studies have already shown that the performance of CAS is comparable to manual screening, although they differ in their test characteristics (specificity and sensitivity) [2]. While many studies found increased sensitivity for ASC‐US and higher (ASCUS+) for CAS compared to manual screening, some studies also found lower sensitivity which was outbalanced by the increased productivity in terms of cost‐effectiveness. Furthermore, Palmer *et al*. showed a broad variety in sensitivity, specificity and positive predictive value of both CAS and manual screening in‐between laboratories [5]. The learning curve and experience of lab technicians with the technology might therefore be an important factor for the performance of CAS. Under what circumstances CAS will be cost‐effective in a primary HPV‐based screening programme compared to manual cytology triage has yet to be investigated.

The aim of this study was to evaluate the circumstances under which CAS will be cost‐effective in a primary hrHPV‐based cervical cancer screening programme compared to the current manual cytology triage screening strategy. The circumstances that influence cost‐effectiveness include both changing costs (due to increased productivity and/or reduced workforce) and the probability of detecting (pre‐invasive) cervical lesions of CAS.

## 2 Method

In this study, the CAS method ThinPrep Imaging System (Hologic Corporation, Marlborough, MA, USA) was used. CAS was compared to the current Dutch manual screening situation to evaluate under which circumstances CAS will be cost‐effective compared to manual cytology triage, using the MIcrosimulation SCreening Analysis‐Cervix (MISCAN‐Cervix) model [6].

### 2.1 Dutch Cervical Screening Programme

The current Dutch cervical cancer screening programme entails primary hrHPV screening, which was implemented in 2017 [1]. Women are invited every 5 years between ages 30 and 60. Those who test hrHPV negative at ages 40 and 50 are offered a 10‐year screening interval. Women who test hrHPV positive at age 60 are invited for an extra screening round at age 65. Initially, a hrHPV test is performed, either by self‐sampling or by a general practitioner (GP). Those who used self‐sampling and tested hrHPV positive are referred to the GP for reflex triage cytology. For women who went to the GP to get a sample taken and test hrHPV positive, the triage cytology will be performed on the same sample. Samples are collected in a ThinPrep medium, and subsequently transported to laboratories and analysed by lab technicians or pathologists. HrHPV‐positive women with high‐grade squamous intra‐epithelial lesions (HSIL) or worse are directly referred to colposcopy. Women with low‐grade cytological abnormalities are referred for repeat cytology after 6 months. Recently, a new triage method was implemented in the screening programme by changing the cut‐off value for referral to colposcopy (instead of referring all HPV and cytology‐positive women to colposcopy, only HPV16/18 and cytology‐positive women are referred) and by extending the interval for control cytology from 6 to 12 months. However, all analyses, in the current study, were performed following the old triage method (see flowchart Figure S1). Since July 2023, the CAS method ThinPrep Imaging System has been used in a fixed proportion of the samples (10%–20%) after a pilot study. Results of this pilot study were not known when performing the current study.

### 2.2 MISCAN‐Cervix

To estimate the effects of using CAS, we conducted an analysis using the MISCAN‐Cervix microsimulation model. MISCAN‐Cervix is a well‐documented semi‐Markov microsimulation software programme. Details of the model have been described before [7]. In short, the model simulates individual life histories of a population based on Dutch population data (i.e., birth rates, life expectancy and cervical cancer epidemiology), in which women may obtain one or more HPV infections. These HPV infections may clear or progress to precancerous lesions (cervical intraepithelial neoplasia [CIN]). The lesions may regress or develop into cervical cancer classified in FIGO (International Federation of Gynaecology and Obstetrics) stages 1A, 1B, 2, 3 and 4. HPV infections are categorised into four groups based on their oncogenicity and presence in different types of HPV vaccines (i.e., bi‐, quadri‐ and non‐avalent vaccines): (I) HPV‐16, (II) HPV‐18, (III) HPV‐31/33/45/52/58 and (IV) HPV‐35/39/51/56/59/66/68. The lesions’ progression probability is dependent on the HPV‐type category, age and lesion grade (CIN1/2/3). Precancerous lesions can develop without HPV, but cervical cancer can only develop in the presence of HPV. However, not all cancers are detected by the HPV test due to a lack of sensitivity (Table S1). Death can occur from either cervical cancer or from other causes. Life histories of women can be affected by screening interventions. A cohort of 10 million women born in 1992 was simulated with the MISCAN model. The effects of screening on cancer incidence and mortality were estimated in this simulated population.

### 2.3 Model Assumptions

#### 2.3.1 Screening Strategies

Women undergo primary HPV screening with either (full) manual cytology or CAS triage during their lifetime between ages 30 and 60 according to the current Dutch screening programme and are followed from birth until death. We simulated 198 different scenarios with varying probabilities to detect a CIN1 (−10% to +10%, with 2% interval; i.e., in‐ or decreased CIN2+ specificity) and CIN3 (0% to +4% with 2% interval; i.e., increased CIN3+ sensitivity) and reductions in costs per test (−10 to 0 Euros (€), with an interval of €2) of the current screening programme (see Table S2). Dutch population (i.e., HPV‐prevalence, detection rates, cancer incidence and stage distributions) and screening data (i.e., test characteristics of the HPV test, cytology test and colposcopy, attendance rates per age category and sampling method) were used to estimate the effects of screening. Population‐based observed age‐specific attendance rates (period 2018) of the current programme were used for all scenarios.

#### 2.3.2 Test Characteristics

The assumed sensitivity and specificity of the hrHPV test and manual cytology triage are presented in Table S1. For the current (manual) screening strategy, the probability of detecting a CIN1 and CIN3 were assumed to be 46.1% and 96.1%, respectively, based on Dutch population and screening data (Table S2). For the CAS screening modality, different probabilities to detect cervical lesions were assumed based on the literature and expert opinion. Data were extracted from the most recently published literature review that summarised the performance of CAS compared to manual screening of all articles published in the last 15 years [8]. From this review, we used the articles comparing the ThinPrep Imaging System method with manual screening and extracted or calculated the probabilities to detect cervical lesions (CIN1/2/3) from each usable individual article. After consultation with an expert in the field due to heterogeneous results in the literature, we decided to vary the probability of detecting CIN1 for CAS ranging from −10% to +10% with intervals of 2%, compared to the probability of detecting a CIN1 of the current programme (see Table S2). Previous articles reported a higher sensitivity of CAS for high‐grade lesions (i.e., CIN3), so the probability of detecting CIN3 was varied between 0% and 4% with intervals of 2%. This maximum of 4% increase is the result of the model assumption, based on previous model calibrations, that 12% of the lesions will be systematically missed (i.e., 0% sensitivity), and the remaining 88% of the lesions have a 96% probability to be detected in case of manual cytology triage [7]. Thus, we assumed that this latter probability can increase up to 100% (i.e., a maximum increase of 4%) in case of CAS.

#### 2.3.3 Costs and Disutilities

Costs include screening costs (costs of sending invitations, screening tests, GP sampling and cytological evaluation) and costs of diagnosis and treatment (colposcopy, biopsy, histology, treatment and palliative care) (Table S3). All costs were obtained from the National Institute for Public Health and the Environment and from cost studies [9, 10]. For CAS, costs were based on reductions in screening time based on the literature and expert opinion. In the literature, reductions in screening time were found ranging from 37.5% to 55% [11, 12, 13, 14]. Based on these reductions, the potential personnel savings were calculated (Table S4). Cost reductions were varied from €0 to €10 with intervals of €2, compared to the cytology costs of the current manual screening programme used in MISCAN (Table S2). Utilities were obtained from a Dutch study [15].

### 2.4 Cost‐Effectiveness Analysis

The total costs and (quality‐adjusted) life years ((QA)LYs) gained due to screening (both manual cytology triage and CAS) were calculated. A cost‐effectiveness analysis was conducted with discounted future costs, QALYs and LYs using an annual rate of 3%, towards the year 2022 (i.e., when all women are age 30). Primary outcomes were the average cost‐effective ratios (ACERs) of different scenarios compared to a scenario without screening, in terms of costs per QALY gained and costs per LY gained. Outcomes for CAS scenarios were then compared to the current manual cytology triage screening scenario.

The change in costs per (QA)LY gained per CAS scenario compared to manual cytology triage is presented in a heat map. All CAS strategies with a negative CER are considered to be more cost‐effective than the current manual cytology triage screening strategy. The MISCAN‐Cervix model and all analyses were coded in Python version 3.10.

### 2.5 Sensitivity Analyses

A sensitivity analysis was performed to assess the robustness of our results when our assumptions for discount rates varied. Instead of the international rate of 3%, we applied the Dutch recommended annual rate of 4%, 1.5% and 1.5% for discounting future costs, QALYs and LYs, respectively. These rates are based on the guidelines of the National Health Care Institute in the Netherlands [16].

## 3 Results

### 3.1 Effectiveness and Costs of Screening

Compared to no screening, the current strategy of manual cytology triage for hrHPV‐positive women results in a gain of 2288 QALYs per 100,000 women. When comparing CAS to manual cytology triage, the variation in QALYs gained ranges from a decrease of 10 QALYs (or −0.3%) in scenarios assuming a 4% decrease in the probability of detecting CIN1 and a 2% increase in detecting CIN3, to an increase of 6 QALYs (or +0.3%) in scenarios with a 10% decrease in detecting CIN1 and no change in the probability of detecting CIN3, per 100,000 women born (see Table S5).

Compared to no screening, the current strategy of manual cytology triage for hrHPV‐positive women leads to a gain of 1626 life years (LYs) per 100,000 women. When CAS is used instead of manual cytology triage, the variation in LYs gained ranges from a decrease of 16 LYs (or −1.0%) in the scenario assuming a 6% decreased probability of detecting CIN1 with no change in the probability of detecting CIN3, to an increase of 20 LYs (or +1.2%) in the scenario with a 10% increased probability of detecting CIN1 and a 4% increased probability of detecting CIN3, per 100,000 women born (see Table S6).

Compared to no screening, the total costs of manual cytology triage for hrHPV‐positive women are €209 per woman. When comparing CAS to manual cytology triage, the difference in lifetime costs ranges from a reduction of €9 (or −4.3%) in scenarios assuming a 10% decreased probability of detecting CIN1 with no change in detecting CIN3 and a cost reduction of €10, to an increase of €3 (or +1.4%) in scenarios where there is a 10% increased probability of detecting CIN1 with no change in detecting CIN3, per woman (see Table S7).

### 3.2 Cost‐Effectiveness

Table 1 presents the difference in cost‐effectiveness ratios (CERs; costs per QALY gained) for the different CAS scenarios compared to current, manual cytology triage. Compared to current screening, CAS will be more cost‐effective in all scenarios, except for the following combinations: (I) when costs are equal to manual cytology triage and an increased probability of detecting CIN1 (up until +10%; i.e., decreased CIN2+ specificity), (II) a cost reduction of €2 and an increased probability of detecting CIN1 from +4% onwards or (III) a cost reduction of €4 and an increased probability of detecting CIN1 from +6% onwards. All CAS scenarios with any reduction in the probability of detecting CIN1 (i.e., increased CIN2+ specificity), despite cost reduction or the change in probability of detecting CIN3 seem to be a cost‐effective strategy compared to manual cytology triage. Also, CAS is always more cost‐effective than manual testing in case of a cost reduction of ≥ €6 compared to manual cytology triage. The most cost‐effective scenario (CER: −€420/QALY gained) is the scenario with the largest decrease in the probability of detecting a CIN1 (−10%) and the largest reduction in costs per test (−€10). When using LYs instead of QALYs as an outcome, a similar pattern is observed (Table 2). All scenarios with any reduction in the probability of detecting a CIN1 lesion or a cost‐reduction from €4 onwards seem to be cost‐effective, compared to manual cytology triage.

**TABLE 1:** Difference in costs (in €) per quality of life year gained of computer‐assisted screening (CAS) scenarios compared to manual screening, using international discount rates of 3% for QALYs and costs.

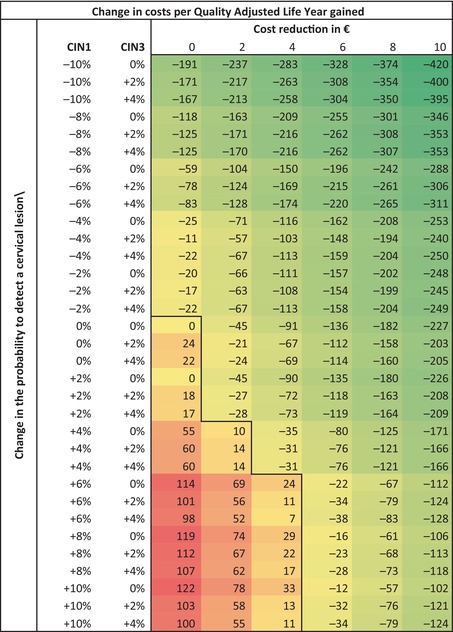

*Note:* The black line indicates the boundary between CAS scenarios that are cost‐effective (in green) and scenarios that are not cost‐effective (in red) compared to manual screening.

**TABLE 2:** Difference in costs (in €) per life year gained of computer‐assisted screening (CAS) scenarios compared to manual screening, using international discount rates of 3% for LY and costs.

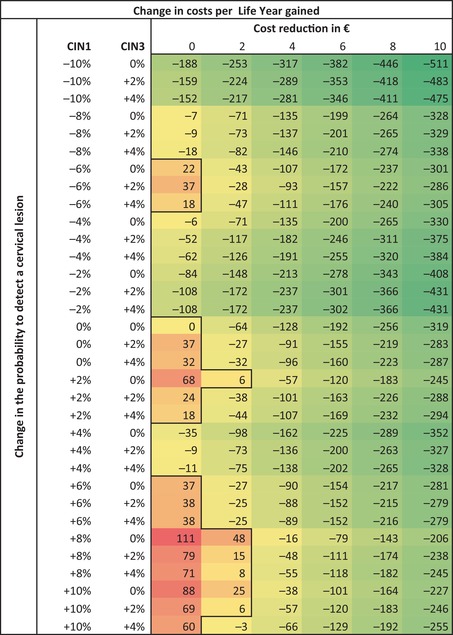

*Note:* The black line indicates the boundary between CAS scenarios that are cost‐effective (in green) and scenarios that are not cost‐effective (in red) compared to manual screening.

### 3.3 Sensitivity Analysis

Using an annual discount rate of 1.5% for effects and 4% for costs instead of 3%, resulted in similar CAS scenarios that were cost‐effective compared to manual cytology triage, but the differences in costs per (QA)LY gained were smaller if we compare CAS to manual cytology triage (Tables S8 and S9).

## 4 Discussion

This study assessed the conditions under which CAS is cost‐effective compared to current manual cytology triage within a primary hrHPV‐based cervical screening programme. The most cost‐effective strategies were found to be strategies with a higher reduction in screening costs and a decreased probability of detecting a CIN1 (i.e., increased CIN2+ specificity). All CAS scenarios with any reduction in the probability of detecting a CIN1 (so, an increased CIN2+ specificity) or a reduction in costs from €6 per test onwards suggested more cost‐effective strategies compared to manual cytology triage. Increased test sensitivity for CIN3 seems not to have a large impact on the costs‐effectiveness of screening, although it may be regarded as positive for the effectiveness of screening.

In literature, only one study investigates the cost‐effectiveness of a computer‐assisted tool for cervical cancer screening, by using artificial intelligence (AI). This Chinese study found that AI‐assisted liquid‐based cytology every 5 years could be more cost‐effective than manual liquid‐based cytology [17]. However, this study used a different method (AI vs. CAS with Thinprep) and only AI‐assisted cytology alone was compared to both manual cytology alone and primary HPV testing with manual cytology triage. Our study analysed the CAS triage in a primary HPV‐based setting (primary HPV test with CAS and manual cytology triage).

Various publications showed heterogeneous results regarding test characteristics of CAS compared to manual screening. In addition, it has been shown that test sensitivity of CAS is highly influenced by the technological experience of cytotechnologists [11, 14, 18]. Studies have shown that greater experience with the technology improves the interpretation of the fields marked by the scanner and, therefore, leads to a higher overall sensitivity of CAS [14]. Moreover, the fact that slides are first screened with CAS and abnormal results are marked, might cause higher awareness of cytotechnologists of the abnormality detected. This could also contribute to a higher test sensitivity of CAS. Training of cytotechnologists will therefore play an important role in the cost‐effectiveness of CAS.

In our study, we showed that a decreased probability of detecting a CIN1 (i.e., increased CIN2+ specificity) resulted in lower costs per (QA)LY gained of CAS, compared to manual cytology triage. This can be explained by the fact that, due to the decreased probability of detecting a CIN1, there will be fewer unnecessary referrals for clinically irrelevant lesions. This means that there will be fewer overdiagnoses, resulting in lower costs and quality of life losses. As CAS will be cost‐effective in all scenarios with any reduction in the probability of detecting a CIN1, compared to manual cytology triage, it will be important to focus on maintaining a good specificity when using CAS. We also showed that the increased probability of detecting CIN3 did not impact the cost‐effectiveness results. This might be the result of the small variation we applied (variation up to 4%) to this parameter of CAS, compared to manual cytology triage, as the sensitivity of CIN3 is already high in the current Dutch cervical screening programme. The impact on the cost‐effectiveness of screening of CAS might be different in other screening settings with lower cytology performance. We were unable to increase the sensitivity further, as it reached 100% due to the 12% of systematically missed CIN lesions (in case of cytology) that are taken into account in the model. These systematically missed lesions are the result of lesions that are located higher up in the cervical canal and therefore are more difficult to detect. Furthermore, the lack of a consistent in‐or decreasing trend in the (QA)LYs gained due to in‐or decreased CIN detection probabilities (Tables S5 and S6) can be explained by random fluctuations in model outcomes.

The improved cost‐effectiveness of CAS is mainly the result of the reduction in screening costs of CAS, compared to manual cytology triage. The loss of QALYs is a result of increased unnecessary referrals for scenarios with decreased CIN2+ specificity of CAS, compared to manual cytology triage. Unnecessary referral may cause anxiety [19] and could potentially lead to overtreatment, contributing to a lower quality of life. This also explains why more scenarios are cost‐effective in terms of LYs gained compared to QALYs gained, as the quality of life impact of unnecessary referrals is not taken into account when considering LYs gained as an outcome.

Since 2023, vaccinated cohorts entered the Dutch cervical cancer screening programme. As the HPV vaccine is relatively more effective against high‐grade lesions [20], it is expected that relatively more CIN1 lesions will be found in vaccinated cohorts. Especially those CIN1 lesions are prone to misclassification and inter‐observer variability, as often normal biopsies are misclassified as CIN1 [21]. Therefore, CAS could offer a solution by providing an objective classification in distinguishing abnormal cells from normal cells. Also, with implementation of CAS, these slides classified as ‘normal’ will save significant screening time and costs as they do not have to be fully inspected. On the other hand, due to the vaccination, it is expected that the HPV16 and HPV18 positivity will be decreased among these vaccinated cohorts and subsequently less cytology might be needed. This may lead to a reduced workload and may lower the urge for CAS. However, a 14‐type HPV DNA test will still detect other oncogenic HPV types and therefore, the decrease in total HPV‐positivity rates and cytology triage tests is expected to be modest. The effectiveness of CAS in a vaccinated population needs to be further examined.

To our knowledge, this is the first study investigating the cost‐effectiveness of CAS compared to manual cytology triage in a primary HPV‐based setting. The results of this study show that CAS will be cost‐effective for any reduction in costs per test compared to manual cytology triage. This scenario is realistic, as the costs of CAS are expected to be lower compared to manual cytology triage, due to the reduction in screen time and therefore increased productivity of CAS. CAS will not be cost‐effective when costs are equal to manual cytology triage and the probability of detecting CIN1 is increased. So, although the costs are expected to be lower compared to manual cytology triage, it is also important not to decrease the CIN2+ specificity. In order to enhance the increased specificity, it will be recommended to have a quality assurance system including training for analysts. Our results can be used as a guideline under which circumstances CAS would be cost‐effective to implement in a national screening programme. Moreover, in the literature, all studies investigating CAS use a cytology‐based screening programme as a reference. In a primary HPV‐based screening programme, the probability of detecting abnormalities is increased because of the higher sensitivity of the HPV test, compared to a cytology‐based screening programme [1]. As more countries are switching to an HPV‐based screening programme [22], it is important to gain more insight into the performance of CAS in a primary HPV‐based screening setting. In countries that employ a primary HPV‐based screening programme complemented by triage methods such as extended HPV genotyping, 7‐type HPV mRNA tests, p16/Ki67 dual staining, or methylation markers, the relevance of triage using CAS may be diminished. Still, although promising, these novel techniques are not yet suitable for population‐wide implementation, and cytology will remain the main triage technique in the upcoming years.

Furthermore, we used a well‐validated and calibrated microsimulation model, which has been updated based on the Dutch population and screening data over years and is used in many studies to inform policymakers for optimising screening programmes [23, 24, 25]. Our study also has several limitations. Firstly, we did not take into account the costs of the system purchase. In laboratories in the Netherlands, the current cytology system is leased. We assumed the costs for leasing the CAS system would be roughly similar to the current depreciation costs. The high upfront costs of investing in these systems might influence the cost‐effectiveness of CAS when such a system is purchased (purchase costs are equal to roughly 1.0 FTE analysis per year). However, in the long term, it is expected that savings from personnel costs and increased productivity could outbalance this once‐in‐a‐lifetime investment. Secondly, in the Netherlands, the cytology triage has a high performance. However, new biomarker triage strategies, such as HPV‐methylation, are being investigated and show promising results without requiring a cytology infrastructure or CAS utilisation [26]. This is especially relevant in the context of HPV self‐sampling, which broadens the accessibility and applicability of these strategies [27]. Currently, as almost all screening programmes in Europe either use cytology as a primary test or as a triage strategy [22], CAS will be valuable for these countries and the results of this study can be used as a guideline. Thirdly, recently (since July 2022), the triage strategy in the Netherlands has been changed from referring all HPV‐positive women with cytology‐positive test results to colposcopy, to only referring HPV16‐ and HPV18‐positive women with cytology‐positive test results to colposcopy. For other (non‐HPV16/18) positive HPV types, and low‐grade cytology results, women are referred to repeat cytology in 12 months (high‐grade cytology is still directly referred). We did not take this new triage strategy into account in our analyses. However, as more women are referred for an additional cytology test, we expect CAS will be relatively more cost‐effective in this new triage setting since CAS reduces the workload of cytology.

In conclusion, our study demonstrates that under realistic conditions, CAS is likely to be cost‐effective compared to manual cytology triage for women with a positive hrHPV test in primary screening. It is crucial to focus on reducing screening costs while maintaining the specificity of CAS. Additionally, the training of cytotechnologists plays a vital role in the successful implementation of CAS. Our findings provide practical guidelines for when CAS may be preferable to manual cytology triage within an organised cervical cancer screening programme.

## Author Contributions

**Ellen M. G. Olthof:** conceptualization (lead), formal analysis (equal), visualization (lead), writing – original draft (lead). **S. Kaljouw:** formal analysis (equal), writing – review and editing (equal). **Folkert J. van Kemenade:** supervision (equal), writing – review and editing (equal). **Anne M. Uyterlinde:** writing – review and editing (equal). **Inge M. C. M. de Kok:** supervision (equal), writing – review and editing (equal).

## Ethics Statement

The authors have nothing to report.

## Conflicts of Interest

The authors declare no conflicts of interest.

## Supporting information


Data S1.

## Data Availability

Detailed modelling results are available upon reasonable request.
