# Supplementary material for: Cost‐Effectiveness of Computer‐Assisted Cytology in a Primary hrHPV‐Based Cervical Cancer Screening Programme
Source: Cancer Med. 2024 Oct 14;13(19):e70299. doi: 10.1002/cam4.70299 (PMC11472647; doi:10.1002/cam4.70299)
Supplement: Supplementary file 1 — Data S1. [file CAM4-13-e70299-s001.docx]

# AppendiCES

*Supplement to: EMG Olthof, S Kaljouw, FJ van Kemenade, AM Uyterlinde and IMCM de Kok. Cost-effectiveness of computer-assisted cytology in a primary hrHPV-based cervical cancer screening programme.*

**Appendix A: The screening programme simulated in MISCAN**

**Figure S1:** Flowchart of the Dutch cervical cancer screening programme……………**2**

**Appendix B: Assumptions for test characteristics and costs of screening 3**

**Table S1:** Test characteristics of the hrHPV- and cytology test by disease status

**Table S2:** Assumptions of test characteristics and costs for manual screening and CAS

**Table S3**: Costs and Disutilities of screening of current manual screening

**Table S4:** Calculation of the difference in screening costs between CAS and current manual screening, assuming a screening time reduction of 37.5% and 55.0% with CAS

**Appendix C: Results of the costs and effects of screening 7**

**Table S5** The difference in QALYs per 100.000 women of computer assisted screening scenarios compared to manual screening, using discount rates of 3% for QALYs.

**Table S6** The difference in LYs per 100.000 women of computer assisted screening scenarios compared to manual screening, using discount rates of 3% for LYs.

**Table S7** The difference in lifetime costs per woman of computer assisted screening scenarios compared to manual screening, using discount rates of 3% for costs.

**Appendix D: Results from sensitivity analyses 9**

**Table S8** Results of the sensitivity analysis. Difference in costs per quality of life year gained of computer assisted screening scenarios compared to manual screening, using discount rates of 1.5% for QALYs and 4% for costs.

**Table S9** Results of the sensitivity analysis. Difference in costs per life year gained of computer assisted screening scenarios compared to manual screening, using discount rates of 1.5% for LY and 4% for costs.

**Appendix A: The screening programme simulated in MISCAN**


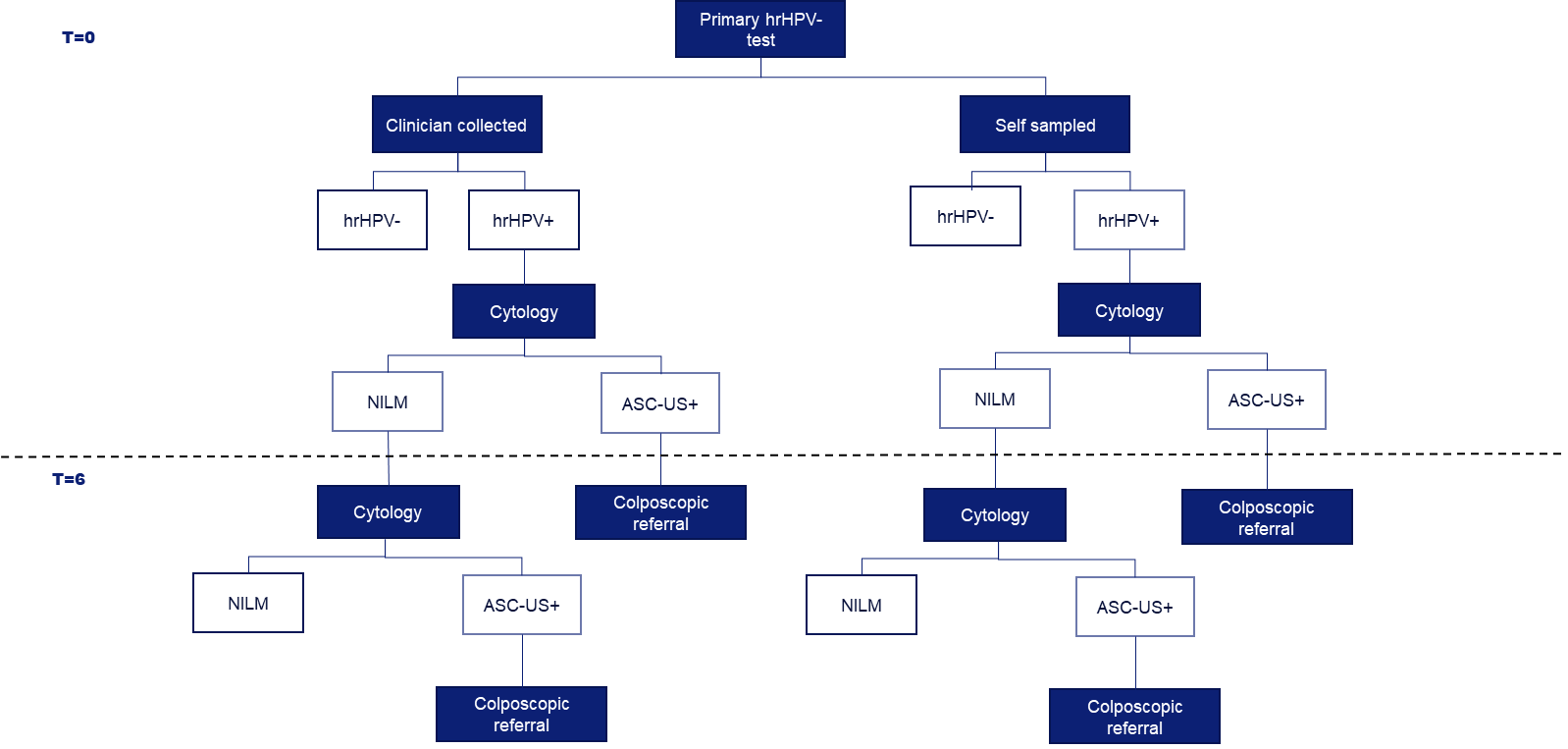


**Figure S1** Flowchart of the Dutch cervical cancer screening programme

**Appendix B: Assumptions for test characteristics and costs of screening**

**Table S1:** Test characteristics of the hrHPV- and cytology test by disease status

| Test result and disease status | Probability of a positive test result |
| --- | --- |
| *Cytology ≥ASC-US in case of no prevalent hrHPV infection** |  |
| - No CIN present | 0.60% |
| - CIN1 | 41.24% |
| - CIN2 | 42.25% |
| - CIN3 | 85.80% |
| - Cervical cancer | 85.09% |
| *Cytology ≥ASC-US in case of ≥1 prevalent hrHPV infection** |  |
| - No CIN present | 17.08% |
| - CIN1 | 41.24% |
| - CIN2 | 42.25% |
| - CIN3 | 85.80% |
| - Cervical cancer | 85.09% |
| *Cytology ≥HSIL in case of no prevalent hrHPV infection** |  |
| - No CIN present | 0.04% |
| - CIN1 | 2.98% |
| - CIN2 | 12.19% |
| - CIN3 | 58.73% |
| - Cervical cancer | 64.73% |
| *Cytology ≥HSIL in case of ≥1 prevalent hrHPV infection** |  |
| - No CIN present | 0.00% |
| - CIN1 | 2.98% |
| - CIN2 | 12.19% |
| - CIN3 | 58.73% |
| - Cervical cancer | 64.73% |
| *Positive hrHPV-test**, in case of no prevalent hrHPV infection* | 0% |
| *Positive hrHPV-test**, in case of ≥1 prevalent hrHPV infection* |  |
| - No CIN present | 55% |
| - CIN1 | 72% |
| - CIN2 | 94% |
| - CIN3 | 94% |
| - Cervical cancer | 94% |

* Probability to test positive the first time a women with this lesion present attends screening. 12% of the CIN lesions will be missed systematically over time.

**The same test characteristics are assumed for GP smears as for self-sampling kits

hrHPV = high-risk human papillomavirus; CIN = cervical intraepithelial neoplasia; ASC-US = Atypical squamous cells of undetermined significance; LSIL = Low-grade squamous intraepithelial lesion; HSIL = High-grade squamous intraepithelial lesion

| **Parameter** | **Range** | **Interval** | **Manual screening*** | **CAS range*** |
| --- | --- | --- | --- | --- |
| Probability to detect a CIN1 | -10% to +10% | 2% | 46.1% | 36.1% to 56.1% |
| Probability to detect a CIN3 | +0% to +4% | 2% | 96.1% | 96.1% to 100.1% |
| Cytology costs |  |  |  |  |
| Reflex cytology after CC | -€10 to €0 | €2 | €26 | €16 to €26 |
| Reflex cytology after SS | -€10 to €0 | €2 | €52 | €42 to €52 |
| Repeat cytology | -€10 to €0 | €2 | €53 | €43 to €53 |

**Table S2:** Assumptions of test characteristics and costs for manual screening and CAS

*These sensitivities are not adjusted for systematic errors and are therefore not total sensitivities. The average total sensitivity will be lower because of the correction for systematic errors (which is assumed to be 12%) which can be attributed to the location of the lesion.

**Table S3**: Costs and disutilities of current manual screening

|  | Laboratory costs | Total screening costs | Disutility’s | Duration |
| --- | --- | --- | --- | --- |
|  | (€) | (€) | (year) | (months) |
| **SCREENING** |  |  |  |  |
| Primary hrHPV-test | 16 | 58 | 0 | 0 |
| Primary hrHPV self-sampling | 14 | 43 | 0 | 0 |
| Reflex manual cytology after hrHPV-test | 26 | 26 | 0 | 0 |
| Repeat manual cytology after hrHPV self-sampling | 33 | 52 | 0.03 | 1 |
| Repeat manual cytology after 6 months | 33 | 53 | 0.03 | 6 |
| **DIAGNOSIS AND TREATMENT** |  |  |  |  |
| No CIN |  | 316 | 0.03 | 1 |
| CIN1 |  | 986 | 0.03 | 1 |
| CIN2 |  | 1 461 | 0.03 | 1 |
| CIN3 |  | 1 710 | 0.03 | 1 |
| FIGO1A |  | 5 601 | 0.08 | 12 |
| FIGO1B |  | 13 283 | 0.08 | 12 |
| FIGO2+ Clinically detected |  | 12 226 | 0.14 | 12 |
| FIGO2+ screen detected |  | 13 092 | 0.14 | 12 |
| Surviving cancer |  | 0 | 0.03 | 120 |
| Palliative care |  | 29 745 | 0.5 | 12 |

**Table S4:** Calculation of the difference in screening costs between CAS and current manual screening, assuming a screening time reduction of 37.5% and 55.0% with CAS

|  | **Current manual screening** | |  | **CAS  screening time reduction 37.5%** | | | **CAS  screening time reduction 55%** | | | **Difference Manual - CAS screening time reduction 37.5%** | **Difference Manual - CAS screening time reduction 55%** |
| --- | --- | --- | --- | --- | --- | --- | --- | --- | --- | --- | --- |
|  | Time per slide | Salary per min | Total cost | Time per slide | Salary per min | Total cost | Time per slide | Salary per min | Total cost | Total costs | Total costs |
|  | (min) | (€) | (€) | (min) | (€) | (€) | (min) | (€) | (€) | (€) | (€) |
| **Reflex cytology after hrHPV-test** |  |  |  |  |  |  |  |  |  |  |  |
| Pathologist | 9.94 | 2.01 | 19.98 | 9.94 | 2.01 | 19.98 | 9.94 | 2.01 | 19.98 |  |  |
| Analyst | 16.05 | 0.67 | 10.75 | 10.03 | 0.67 | 6.72 | 7.22 | 0.67 | 4.84 |  |  |
| Total |  |  | **30.73** |  |  | **26.70** |  |  | **24.82** | **-4.03** | **-5.91** |
|  |  |  |  |  |  |  |  |  |  |  |  |
| **Repeat cytology after hrHPV self-sampling** |  |  |  |  |  |  |  |  |  |  |  |
| Pathologist | 9.94 | 2.01 | 19.98 | 9.94 | 2.01 | 19.98 | 9.94 | 2.01 | 19.98 |  |  |
| Analyst | 16.05 | 0.67 | 10.75 | 10.03 | 0.67 | 6.72 | 7.22 | 0.67 | 4.84 |  |  |
| Administrative assistant only with self-sampling 19.9% | 3.8 | 0.52 | 0.39 | 3.8 | 0.52 | 0.39 | 3.8 | 0.52 | 0.39 |  |  |
| Total |  |  | **31.12** |  |  | **27.09** |  |  | **25.21** | **-4.03** | **-5.91** |
|  |  |  |  |  |  |  |  |  |  |  |  |
| **Repeat cytology after 6 months** |  |  |  |  |  |  |  |  |  |  |  |
| Pathologist | 10.93 | 2.01 | 21.97 | 10.93 | 2.01 | 21.97 | 10.93 | 2.01 | 21.97 |  |  |
| Analyst | 18.77 | 0.67 | 12.58 | 10.03 | 0.67 | 6.72 | 7.22 | 0.67 | 4.84 |  |  |
| Administrative assistant | 3.8 | 0.52 | 1.98 | 3.8 | 0.52 | 1.98 | 3.8 | 0.52 | 1.98 |  |  |
| Total |  |  | **36.53** |  |  | **30.67** |  |  | **28.78** | **-5.86** | **-7.75** |

**APPENDIX C: Results of the costs and effects of screening**

**Table S5** The difference in QALYs per 100.000 women of computer assisted screening scenarios compared to manual screening, using discount rates of 3% for QALYs.

| **Total Quality Adjusted Life Years gained of CAS compared to manual screening** | | | | |
| --- | --- | --- | --- | --- |
|  |  | **Change in the probability to detect a cervical lesion grade 3 (CIN3)** | | |
|  |  | 0% | +2% | +4% |
| **Change in the probability to detect a cervical lesion grade 1 (CIN1)** | -10% | 6.4 | 2.9 | 2.2 |
|  | -8% | -2.5 | 0.2 | 0.4 |
|  | -6% | -8.4 | -3.2 | -2.1 |
|  | -4% | -7.7 | -9.9 | -7.8 |
|  | -2% | -2.6 | -3.0 | -2.1 |
|  | 0% | 0.0 | -5.1 | -4.6 |
|  | +2% | 5.9 | 2.7 | 3.0 |
|  | +4% | 0.2 | -0.6 | -0.5 |
|  | +6% | -6.8 | -3.4 | -2.6 |
|  | +8% | -2.7 | -1.1 | 0.0 |
|  | +10% | 0.6 | 5.0 | 5.7 |
| *Compared to no screening, 2288 QALYs are gained with manual screening | | | |  |

**Table S6** The difference in LYs per 100.000 women of computer assisted screening scenarios compared to manual screening, using discount rates of 3% for LYs.

| **Total Life Years gained of CAS compared to manual screening** | | | | |
| --- | --- | --- | --- | --- |
|  |  | **Change in the probability to detect a cervical lesion grade 3 (CIN3)** | | |
|  |  | 0% | +2% | +4% |
| **Change in the probability to detect a cervical lesion grade 1 (CIN1)** | -10% | -6.0 | -8.4 | -9.1 |
|  | -8% | -12.2 | -8.6 | -8.4 |
|  | -6% | -15.6 | -9.6 | -8.4 |
|  | -4% | -12.6 | -13.6 | -11.6 |
|  | -2% | -4.6 | -4.1 | -3.1 |
|  | 0% | 0.0 | -4.0 | -3.5 |
|  | +2% | 8.7 | 6.3 | 6.6 |
|  | +4% | 5.4 | 5.3 | 5.4 |
|  | +6% | 1.3 | 5.6 | 6.4 |
|  | +8% | 8.0 | 10.3 | 11.4 |
|  | +10% | 13.5 | 18.7 | 19.5 |
| *Compared to no screening, 1626 LYs are gained with manual screening | | | | |

**Table S7** The difference in lifetime costs per woman of computer assisted screening scenarios compared to manual screening, using discount rates of 3% for costs.

| **Total costs per lifetime of CAS compared to manual screening** | | | | | | | | |
| --- | --- | --- | --- | --- | --- | --- | --- | --- |
| **Change in sensitivity** |  |  | **Cost reduction in €** | | | | | |
|  | **CIN1** | **CIN3** | 0 | 2 | 4 | 6 | 8 | 10 |
|  | -10% | 0% | -3.81 | -4.86 | -5.91 | -6.95 | -8.00 | -9.05 |
|  | -10% | +2% | -3.66 | -4.71 | -5.76 | -6.80 | -7.85 | -8.90 |
|  | -10% | +4% | -3.62 | -4.67 | -5.72 | -6.76 | -7.81 | -8.86 |
|  | -8% | 0% | -2.92 | -3.96 | -5.01 | -6.05 | -7.10 | -8.15 |
|  | -8% | +2% | -2.85 | -3.89 | -4.94 | -5.98 | -7.03 | -8.07 |
|  | -8% | +4% | -2.82 | -3.86 | -4.91 | -5.95 | -7.00 | -8.04 |
|  | -6% | 0% | -2.10 | -3.15 | -4.19 | -5.24 | -6.28 | -7.32 |
|  | -6% | +2% | -2.08 | -3.12 | -4.17 | -5.21 | -6.25 | -7.30 |
|  | -6% | +4% | -2.09 | -3.13 | -4.17 | -5.22 | -6.26 | -7.30 |
|  | -4% | 0% | -1.27 | -2.32 | -3.36 | -4.40 | -5.44 | -6.49 |
|  | -4% | +2% | -1.16 | -2.20 | -3.24 | -4.28 | -5.33 | -6.37 |
|  | -4% | +4% | -1.21 | -2.25 | -3.29 | -4.33 | -5.37 | -6.42 |
|  | -2% | 0% | -0.70 | -1.74 | -2.78 | -3.82 | -4.86 | -5.90 |
|  | -2% | +2% | -0.67 | -1.71 | -2.75 | -3.79 | -4.83 | -5.87 |
|  | -2% | +4% | -0.69 | -1.72 | -2.76 | -3.80 | -4.84 | -5.88 |
|  | 0% | 0% | 0.00 | -1.04 | -2.08 | -3.12 | -4.15 | -5.19 |
|  | 0% | +2% | 0.08 | -0.96 | -1.99 | -3.03 | -4.07 | -5.11 |
|  | 0% | +4% | 0.08 | -0.96 | -2.00 | -3.04 | -4.08 | -5.11 |
|  | +2% | 0% | 0.54 | -0.49 | -1.53 | -2.57 | -3.60 | -4.64 |
|  | +2% | +2% | 0.66 | -0.38 | -1.41 | -2.45 | -3.48 | -4.52 |
|  | +2% | +4% | 0.67 | -0.37 | -1.40 | -2.44 | -3.48 | -4.51 |
|  | +4% | 0% | 1.29 | 0.25 | -0.78 | -1.82 | -2.85 | -3.88 |
|  | +4% | +2% | 1.31 | 0.28 | -0.76 | -1.79 | -2.83 | -3.86 |
|  | +4% | +4% | 1.31 | 0.28 | -0.75 | -1.79 | -2.82 | -3.86 |
|  | +6% | 0% | 1.98 | 0.95 | -0.09 | -1.12 | -2.15 | -3.18 |
|  | +6% | +2% | 2.00 | 0.97 | -0.06 | -1.10 | -2.13 | -3.16 |
|  | +6% | +4% | 1.99 | 0.96 | -0.08 | -1.11 | -2.14 | -3.17 |
|  | +8% | 0% | 2.47 | 1.44 | 0.41 | -0.62 | -1.65 | -2.68 |
|  | +8% | +2% | 2.45 | 1.42 | 0.39 | -0.63 | -1.66 | -2.69 |
|  | +8% | +4% | 2.45 | 1.42 | 0.39 | -0.64 | -1.67 | -2.70 |
|  | +10% | 0% | 2.86 | 1.83 | 0.80 | -0.23 | -1.26 | -2.29 |
|  | +10% | +2% | 2.81 | 1.79 | 0.76 | -0.27 | -1.30 | -2.32 |
|  | +10% | +4% | 2.82 | 1.79 | 0.76 | -0.27 | -1.29 | -2.32 |
| *Compared to no screening, the total costs of manual screening are €209 | | | | | | |  |  |

**APPENDIX D: Results from sensitivity analyses**

**Table S8** Results of the sensitivity analysis. Difference in costs per quality of life year gained of computer assisted screening scenarios compared to manual screening, using discount rates of 1.5% for QALYs and 4% for costs.

| **Change in costs per Quality Adjusted Life Year gained** | | | | | | | | |
| --- | --- | --- | --- | --- | --- | --- | --- | --- |
| **Change in the probability to detect a cervical lesion** |  |  | **Cost reduction in €** | | | | | |
|  | **CIN1** | **CIN3** | 0 | 2 | 4 | 6 | 8 | 10 |
|  | -10% | 0% | -95 | -121 | -147 | -173 | -198 | -224 |
|  | -10% | +2% | -85 | -111 | -137 | -163 | -188 | -214 |
|  | -10% | +4% | -83 | -109 | -134 | -160 | -186 | -212 |
|  | -8% | 0% | -58 | -83 | -109 | -135 | -161 | -187 |
|  | -8% | +2% | -62 | -88 | -114 | -140 | -165 | -191 |
|  | -8% | +4% | -62 | -88 | -114 | -139 | -165 | -191 |
|  | -6% | 0% | -26 | -52 | -78 | -104 | -129 | -155 |
|  | -6% | +2% | -37 | -63 | -89 | -114 | -140 | -166 |
|  | -6% | +4% | -40 | -66 | -91 | -117 | -143 | -169 |
|  | -4% | 0% | -11 | -36 | -62 | -88 | -114 | -139 |
|  | -4% | +2% | -4 | -29 | -55 | -81 | -106 | -132 |
|  | -4% | +4% | -9 | -35 | -61 | -87 | -112 | -138 |
|  | -2% | 0% | -9 | -35 | -60 | -86 | -112 | -137 |
|  | -2% | +2% | -7 | -33 | -58 | -84 | -110 | -135 |
|  | -2% | +4% | -10 | -35 | -61 | -87 | -112 | -138 |
|  | 0% | 0% | 0 | -26 | -51 | -77 | -102 | -128 |
|  | 0% | +2% | 13 | -13 | -39 | -64 | -90 | -115 |
|  | 0% | +4% | 11 | -14 | -40 | -65 | -91 | -117 |
|  | +2% | 0% | -1 | -27 | -52 | -78 | -103 | -129 |
|  | +2% | +2% | 8 | -17 | -43 | -68 | -94 | -119 |
|  | +2% | +4% | 8 | -18 | -43 | -69 | -94 | -120 |
|  | +4% | 0% | 28 | 3 | -23 | -48 | -74 | -99 |
|  | +4% | +2% | 29 | 4 | -22 | -47 | -72 | -98 |
|  | +4% | +4% | 29 | 4 | -22 | -47 | -72 | -98 |
|  | +6% | 0% | 60 | 34 | 9 | -17 | -42 | -68 |
|  | +6% | +2% | 52 | 27 | 1 | -24 | -50 | -75 |
|  | +6% | +4% | 50 | 24 | -1 | -27 | -52 | -77 |
|  | +8% | 0% | 60 | 34 | 9 | -16 | -42 | -67 |
|  | +8% | +2% | 55 | 30 | 4 | -21 | -46 | -72 |
|  | +8% | +4% | 52 | 27 | 2 | -24 | -49 | -74 |
|  | +10% | 0% | 62 | 36 | 11 | -14 | -39 | -65 |
|  | +10% | +2% | 49 | 24 | -1 | -26 | -52 | -77 |
|  | +10% | +4% | 48 | 22 | -3 | -28 | -53 | -78 |

*The black line indicates the boundary between CAS scenarios that are cost-effective (in green) and scenarios that are not cost-effective (in red) compared to manual screening

**Table S9** Results of the sensitivity analysis. Difference in costs per life year gained of computer assisted screening scenarios compared to manual screening, using discount rates of 1.5% for LY and 4% for costs.

| **Change in costs per Life Year gained** | | | | | | | | |
| --- | --- | --- | --- | --- | --- | --- | --- | --- |
| **Change in the probability to detect a cervical lesion** |  |  | **Cost reduction in €** | | | | | |
|  | **CIN1** | **CIN3** | 0 | 2 | 4 | 6 | 8 | 10 |
|  | -10% | 0% | -90 | -123 | -156 | -189 | -222 | -255 |
|  | -10% | +2% | -77 | -110 | -143 | -176 | -209 | -242 |
|  | -10% | +4% | -74 | -107 | -140 | -172 | -205 | -238 |
|  | -8% | 0% | -43 | -76 | -109 | -142 | -175 | -208 |
|  | -8% | +2% | -54 | -86 | -119 | -152 | -185 | -218 |
|  | -8% | +4% | -54 | -87 | -119 | -152 | -185 | -218 |
|  | -6% | 0% | -5 | -38 | -71 | -104 | -137 | -170 |
|  | -6% | +2% | -26 | -59 | -91 | -124 | -157 | -190 |
|  | -6% | +4% | -30 | -63 | -96 | -129 | -161 | -194 |
|  | -4% | 0% | 7 | -26 | -59 | -92 | -125 | -157 |
|  | -4% | +2% | 14 | -19 | -51 | -84 | -117 | -150 |
|  | -4% | +4% | 5 | -28 | -60 | -93 | -126 | -159 |
|  | -2% | 0% | -4 | -36 | -69 | -102 | -134 | -167 |
|  | -2% | +2% | -3 | -36 | -68 | -101 | -133 | -166 |
|  | -2% | +4% | -7 | -40 | -72 | -105 | -138 | -170 |
|  | 0% | 0% | 0 | -32 | -65 | -97 | -130 | -162 |
|  | 0% | +2% | 17 | -16 | -48 | -81 | -113 | -146 |
|  | 0% | +4% | 15 | -18 | -50 | -83 | -115 | -148 |
|  | +2% | 0% | -14 | -46 | -78 | -111 | -143 | -175 |
|  | +2% | +2% | -1 | -34 | -66 | -98 | -131 | -163 |
|  | +2% | +4% | -2 | -35 | -67 | -99 | -131 | -164 |
|  | +4% | 0% | 21 | -11 | -44 | -76 | -108 | -140 |
|  | +4% | +2% | 21 | -11 | -43 | -76 | -108 | -140 |
|  | +4% | +4% | 21 | -11 | -44 | -76 | -108 | -140 |
|  | +6% | 0% | 59 | 26 | -6 | -38 | -71 | -103 |
|  | +6% | +2% | 44 | 11 | -21 | -53 | -85 | -117 |
|  | +6% | +4% | 40 | 8 | -25 | -57 | -89 | -121 |
|  | +8% | 0% | 48 | 16 | -16 | -49 | -81 | -113 |
|  | +8% | +2% | 38 | 6 | -26 | -58 | -90 | -122 |
|  | +8% | +4% | 34 | 2 | -30 | -62 | -94 | -126 |
|  | +10% | 0% | 42 | 10 | -22 | -54 | -85 | -117 |
|  | +10% | +2% | 20 | -11 | -43 | -75 | -107 | -139 |
|  | +10% | +4% | 18 | -14 | -46 | -78 | -110 | -141 |

*The black line indicates the boundary between CAS scenarios that are cost-effective (in green) and scenarios that are not cost-effective (in red) compared to manual screening
